# T2*‐Relaxometry MRI to Assess Third Trimester Placental and Fetal Brain Oxygenation and Placental Characteristics in Healthy Fetuses and Fetuses With Congenital Heart Disease

**DOI:** 10.1002/jmri.29498

**Published:** 2024-07-12

**Authors:** Daniel Cromb, Johannes Steinweg, Jordina Aviles Verdera, Milou P.M. van Poppel, Alexandra F. Bonthrone, David F.A. Lloyd, Kuberan Pushparajah, John Simpson, Reza Razavi, Mary Rutherford, Serena J. Counsell, Jana Hutter

**Affiliations:** ^1^ Centre for the Developing Brain School of Biomedical and Engineering Sciences, King's College London London UK; ^2^ Department of Cardiovascular Imaging School of Biomedical Engineering & Imaging Science, King's College London London UK; ^3^ Biomedical Engineering Department, School of Biomedical Engineering and Imaging Sciences King's College London London UK; ^4^ MRC Centre for Neurodevelopmental Disorders King's College London London UK; ^5^ Smart Imaging Lab, Radiological Institute University Hospital Erlangen Erlangen Germany

**Keywords:** T2*, T2‐relaxometry, placental MRI, fetal brain MRI, congenital heart disease

## Abstract

**Background:**

Congenital heart disease (CHD) has been linked to impaired placental and fetal brain development. Assessing the placenta and fetal brain in parallel may help further our understanding of the relationship between development of these organs.

**Hypothesis:**

1) Placental and fetal brain oxygenation are correlated, 2) oxygenation in these organs is reduced in CHD compared to healthy controls, and 3) placental structure is altered in CHD.

**Study Type:**

Retrospective case–control.

**Population:**

Fifty‐one human fetuses with CHD (32 male; median [IQR] gestational age [GA] = 32.0 [30.9–32.9] weeks) and 30 from uncomplicated pregnancies with normal birth outcomes (18 male; median [IQR] GA = 34.5 [31.9–36.7] weeks).

**Field Strength/Sequence:**

1.5 T single‐shot multi‐echo‐gradient‐echo echo‐planar imaging.

**Assessment:**

Masking was performed using an automated nnUnet model. Mean brain and placental T2* and quantitative measures of placental texture, volume, and morphology were calculated.

**Statistical Tests:**

Spearman's correlation coefficient for determining the association between brain and placental T2*, and between brain and placental characteristics with GA. *P*‐values for comparing brain T2*, placenta T2*, and placental characteristics between groups derived from ANOVA. Significance level *P* < 0.05.

**Results:**

There was a significant positive association between placental and fetal brain T2* (*⍴* = 0.46). Placental and fetal brain T2* showed a significant negative correlation with GA (placental T2* *⍴* = −0.65; fetal brain T2* *⍴* = −0.32). Both placental and fetal brain T2* values were significantly reduced in CHD, after adjusting for GA (placental T2*: control = 97 [±24] msec, CHD = 83 [±23] msec; brain T2*: control = 218 [±26] msec, CHD = 202 [±25] msec). Placental texture and morphology were also significantly altered in CHD (Texture: control = 0.84 [0.83–0.87], CHD = 0.80 [0.78–0.84]; Morphology: control = 9.9 [±2.2], CHD = 10.8 [±2.0]). For all fetuses, there was a significant positive association between placental T2* and placental texture (*⍴* = 0.46).

**Conclusion:**

Placental and fetal brain T2* values are associated in healthy fetuses and those with CHD. Placental and fetal brain oxygenation are reduced in CHD. Placental appearance is significantly altered in CHD and shows associations with placental oxygenation, suggesting altered placental development and function may be related.

**Evidence Level:**

3

**Technical Efficacy:**

Stage 3

The placenta is a vital organ for fetal development, serving as the interface between the maternal and fetal circulations, where it facilitates the exchange of oxygen, nutrients, and waste products. Altered placental structure or function can lead to adverse fetal outcomes, including impaired brain development,^1^ however studies assessing both placental and fetal brain oxygenation in‐vivo have only recently been performed.^2^, ^3^

The MRI transverse relaxation time (T2*‐relaxometry) exploits the blood oxygen level dependent effect. Shorter T2* times are linked to higher concentrations of deoxygenated hemoglobin. It therefore serves as a proxy for tissue oxygenation or function.^4^ Changes in both placental^5^, ^6^ and fetal brain T2*^7^, ^8^ values over gestation have been established in normal pregnancies, and where there is evidence of placental dysfunction,^2^, ^9^ although these are often assessed separately for each organ.

Congenital heart disease (CHD) is the commonest congenital malformation, affecting almost 1% of live births.^10^ It is associated with adverse neurodevelopment spanning multiple domains in almost half of individuals with moderate to severe CHD,^11^ and this impaired neurodevelopment remains one of the greatest problems faced by this population.^12^ Improving understanding of the underlying biological processes associated with altered brain development is crucial to develop interventions aimed at supporting optimal brain development in CHD.

Animal studies revealed that the cardiovascular abnormalities associated with certain types of CHD may compromise cerebral oxygen delivery in‐utero,^13^ and these findings have been supported by human studies.^14^ Previous work has shown that T2* is reduced in the brain of fetuses with moderate to severe CHD.^15^, ^16^ This reduced cerebral oxygenation is likely to contribute to the abnormal brain development seen in fetuses with CHD, including reduced global brain volumes^14^, ^17^, ^18^ and altered cortical development.^19^

Histological examinations have shown that placental development is also affected in CHD^20^ and MRI studies have identified impairments in placental growth^21^ and function in CHD.^22^ This has led to a focus on what has become known as the “heart‐brain‐placenta axis,” acknowledging the complex interplay between these three critical organs, which has particular importance for understanding the impaired brain and placental development seen in CHD.^23^ However, MRI studies investigating placental structure or morphology in‐vivo in normal pregnancies and assessing how these might be affected by CHD are rare.^21^

As a safe, noninvasive imaging method capable of providing quantitative measures of various characteristics of fetal organs, T2* MRI has potential to improve understanding of the link between placental and brain development in‐utero, both in normal pregnancies and those affected by pathology such as CHD.

The aims of this study were to measure placental and fetal brain T2* values in typically developing fetuses and fetuses with CHD to 1) test the hypothesis that placental and fetal brain oxygenation are correlated, 2) test the hypothesis that oxygenation in these organs is reduced in CHD compared to healthy controls, and 3) test the hypothesis that placental structure is altered in CHD.

## Materials and Methods

### Ethical Approval

The National Research Ethics Service West London committee provided ethical approval (07/H0707/105; 17/LO/0292; 14/LO/1806; 18/LO/1958). Informed, written consent was obtained before fetal MRI.

### Subjects

For this retrospective study, all imaging data were acquired between June 2014 and June 2023 at St. Thomas' Hospital, London. For the CHD cohort, women carrying a fetus with serious CHD, confirmed postnatally, who had been referred for a clinical fetal cardiac MRI scan and consented for their images to be used for research were included.

For the control cohort, healthy pregnant women with normal fetal cardiovascular anatomy who agreed to undergo MRI for research were included. For both cohorts, maternal exclusion criteria included multiple pregnancy, chronic hypertension, gestational hypertension, preeclampsia, diabetes or intrauterine growth restriction, weight over 125 kg, severe claustrophobia, inability to give informed consent, or age under 18 years at the time of referral.

Timing of the MRI for the CHD cohort was determined by the clinical team, taking into account gestational age (GA) at diagnosis, the potential impact of new information on fetal counseling and management, and the timeframe for optimal imaging quality, and was not performed before 30 weeks GA. Consequently, only data acquired after 30 weeks GA in the control cohort were included.

**FIGURE 1:**
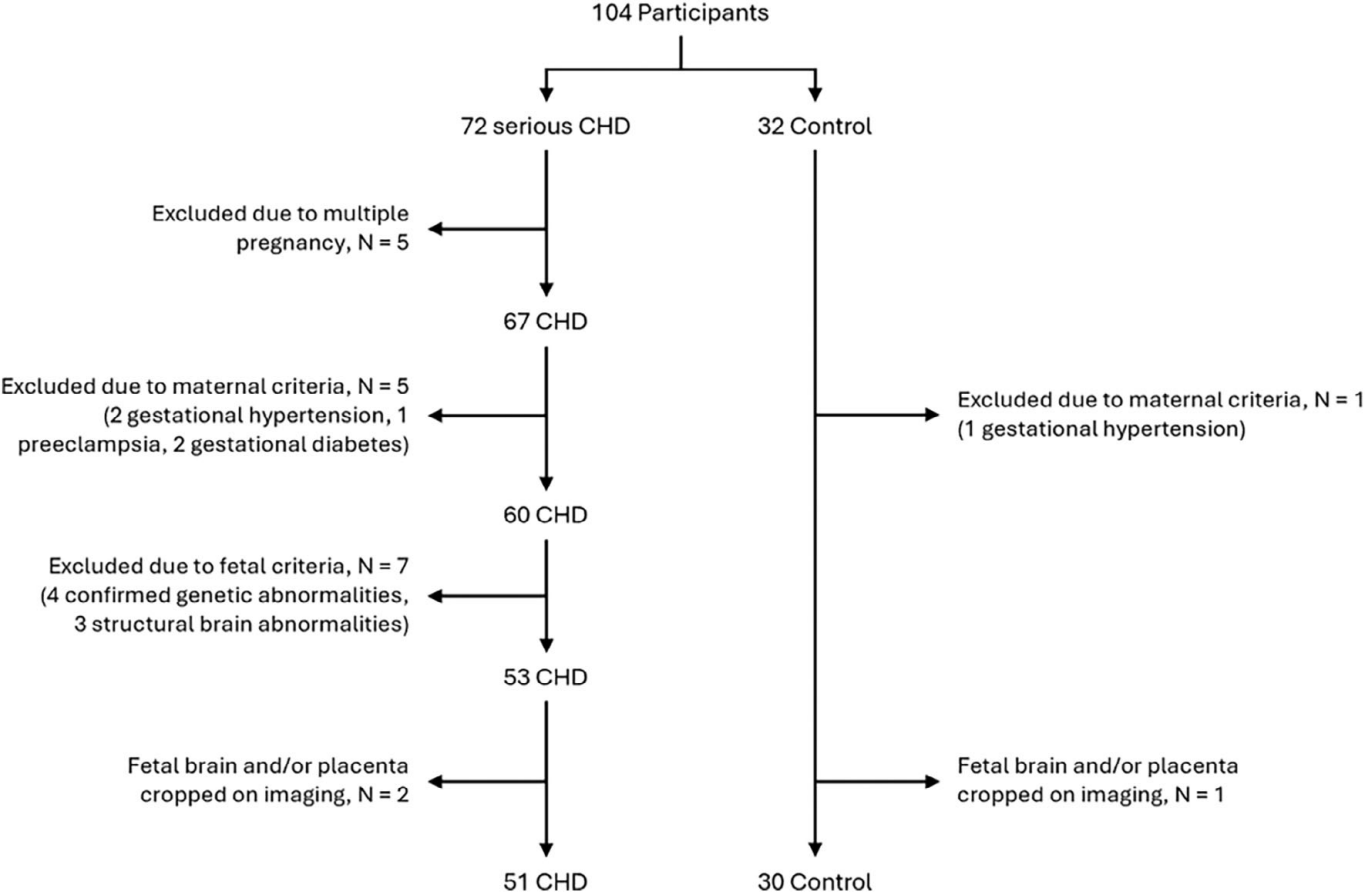
Participant flowchart, highlighting excluded participants for both CHD and control groups. Data for 81 fetuses (30 control and 51 CHD) met the inclusion criteria and were analyzed.

Fetal exclusion criteria included confirmed genetic abnormalities on antenatal or postnatal genetic testing (either from amniocentesis, comparative genomic hybridization array [CGH‐array], or clinical genetic testing) or structural brain abnormalities as reported on fetal MRI, including bilateral ventriculomegaly, cerebellar hypoplasia or absence of the corpus callosum. Poor quality datasets, i.e., those where there was cropping of the placenta or the fetal brain were excluded. Only participants with both placental and fetal brain imaging data meeting all the above criteria were included. Data for 81 fetuses (30 control [18 male; 12 female] and 51 CHD [32 male; 19 female]) met the inclusion criteria and were analyzed (Fig. 1). For this final cohort, median (IQR) GA at scan was 32.4 (31.1–34.0) weeks and median (IQR) maternal age at scan was 32.9 (30.2–36.5) years.

### MR Image Acquisition

Fetal MR images were acquired on a Philips Ingenia 1.5 T scanner, with 28‐channel dStream anterior and posterior in‐built coils. Continuous assessment of maternal blood oxygen saturation levels and heart rate, plus blood pressure measurements at 10‐minute intervals, were performed. As part of the imaging protocol, free‐breathing whole‐uterus multi‐echo single‐shot gradient‐echo echo planar imaging (EPI) data was acquired in coronal orientation to the mother with a resolution of 2.5 mm isotropic, FOV = 360 mm × 360 mm, no sensitivity encoding, no half‐scan, reconstructed matrix size = 224 × 224, WaterFatShift = 11.01, flip angle = 90°, Repetition Time (TR) = 14 seconds, Echo Time (TE) = (11.2, 57.1, 102.9, 148.8, 194.7) milliseconds. Volumetric shimming was applied covering the entire uterus. This multi‐echo sequence was chosen to ensure efficiency and motion robustness with motion effectively being frozen for each slice as all echoes are acquired within <500 milliseconds.

### MR Image Processing

#### AUTOMATIC PREPROCESSING

To ensure reliability of placental and fetal brain T2* measurements, an automated pipeline with no need for manual intervention was used. The data were reconstructed and mono‐exponential fitting was performed in python using least‐square optimization over all available echo times (TEs) as previously reported^24^ to obtain T2* maps for the entire imaging volume using Eq. 1, where *S*(*T*_*E*_) is the signal at the acquired echo times, *T*_*E*_, and *S*_0_ and T2* are the resulting proton density map and T2* map respectively.

(1) $$S\left(T_{E}\right)=S_{0}.e^{\frac{T2*}{T_{E}}}.$$

#### AUTOMATED FETAL BRAIN AND PLACENTAL SEGMENTATION

**FIGURE 2:**
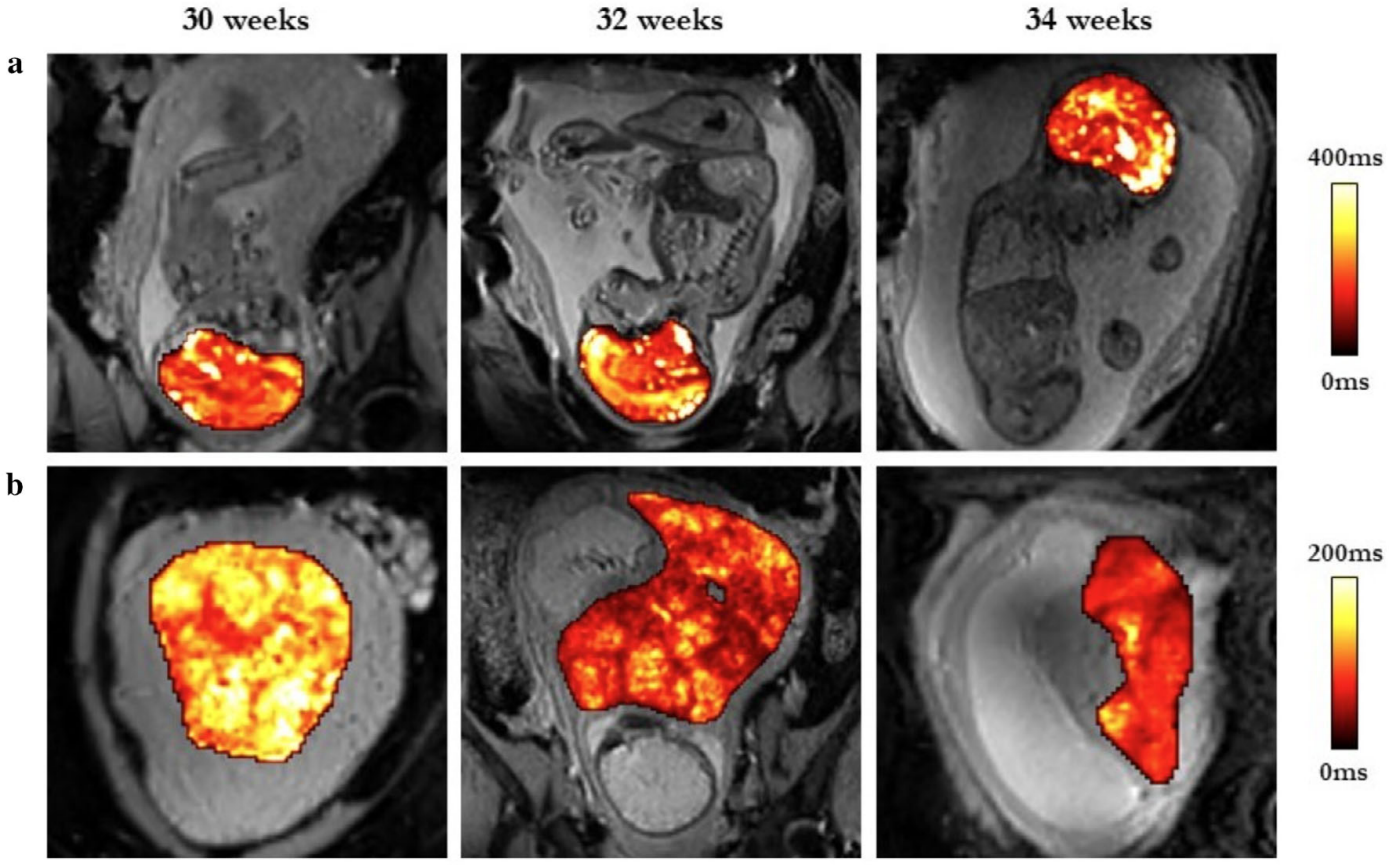
Fetal brain (**a**) and placental (**b**) T2* maps, masked by a bespoke nn‐UNet for the fetal brain and placenta overlaid on gradient‐echo EPI data from six fetuses with CHD imaged for this study.

Two separate neural network‐UNet frameworks that had been trained on gradient‐echo EPI MR data were used to generate automatic segmentations of the placenta and fetal intracranial region. This adaptive semantic segmentation approach is designed to dynamically tailor a segmentation pipeline based on the characteristics of the provided dataset. Through an automated process, it configures a U‐Net‐based segmentation pipeline specifically tailored to the training cases. This automated adaptation removes the need for manual intervention and issues related to inter‐rater reliability for defining regions of interest (ROIs). The network for the fetal intracranial segmentation was trained on a total of 50 cases acquired at 0.55 T and tested on 31 cases acquired at 1.5 T with a mean Dice Score of 0.93. For the placenta network, 210 cases were used (70 at 0.55 T, 70 at 1.5 T, and 70 at 3 T) for the training process and a mean Dice Score of 0.79 was obtained for a testing set of 20 cases at 1.5 T.^5^ Figure 2 shows placental and fetal intracranial masks generated by the network overlaid on T2* maps used for this study.

### Placental and Fetal Brain Characteristics

**FIGURE 3:**
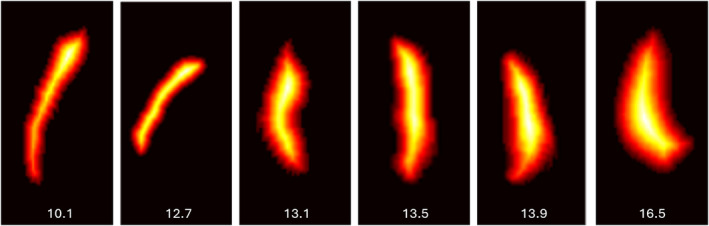
Single mid‐sagittal slices of placental morphological maps for six different placentas, where voxel values are based on their proximity to the mask boundary, thus representing the length of the shortest path from each voxel to the edge of the placenta. Summing the mean and standard deviation of all voxel values in this morphological map allows a unitless measure related to placental morphology to be obtained. An estimate of maximum placental thickness was obtained by multiplying this maximum value in the morphological map by two.

Mean brain and placental T2* values were calculated by averaging all values lower than a threshold of 500 msec, chosen to exclude partial voluming effects from amniotic fluid and cerebrospinal fluid (CSF). Placental volume was calculated by multiplying the number of voxels in the binary placental masks by the voxel dimensions. The texture of placental tissue was evaluated using Grey Level Co‐occurrence Matrix (GLCM) correlation^25^ as part of the Python scikit‐image package, and is referred to from here onwards as *placental texture*. Placental morphology was calculated by first assigning a value to every voxel in the binary placental mask equivalent to its proximity to the mask boundary, and then summing the mean and standard deviation values for all voxels in the resulting map together (Fig. 3). This results in a unitless measure describing the uniformity of placental shape in‐vivo, referred to from here onwards as *placental morphology*. An estimate of maximum placental thickness was obtained by multiplying the highest value in the resulting map by two.

### Statistical Analyses

Statistical analyses were performed using statsmodels (v0.13.2) and Jupyter Notebook, Python3. The Shapiro–Wilk test was used to test normality. Numerical values are presented as mean (±SD) if parametric, or median (interquartile range) if non‐parametric. The Mann–Whitney *U*‐Test was used to test for GA at scan and maternal age at scan differences between groups. A partial Spearman's correlation coefficient was used to determine the direction and significance of the association between fetal brain T2* and placental T2*, after adjusting for GA at scan. Spearman's correlation coefficient was used to determine the direction and significance of any association between fetal brain T2*, placental T2* and all other placental characteristics and GA at scan. An ANCOVA was used to test for differences in fetal brain T2*, placental T2* and placental volume between groups, after adjusting for GA at scan, as these measures showed a significant correlation with GA at scan. Estimated marginal means for fetal brain T2*, placental T2* and placental volume were derived from general linear models with GA at scan included as a covariate. An ANOVA was used to test for differences in placental texture, maximal thickness, and morphology between groups, as these measures showed no significant correlation with GA at scan. Benjamini and Hochberg false discovery rate (FDR) was applied to correct for multiple comparisons (reported as *P*_FDR_). *P*_FDR_‐values <0.05 were considered statistically significant. A post‐hoc analysis using a partial Spearman's correlation analysis was performed to explore the association between placental T2* and placental texture, after accounting for GA at scan. *P*_FDR_‐values <0.05 were considered statistically significant.

## Results

### MR Sequence

Total acquisition time for the whole‐uterus multi‐echo single‐shot gradient‐echo EPI data sequence was less than 1 minute.

### Demographics

GA at scan was significantly higher in the controls group than the CHD group (Controls: 34.5 [31.9–36.7] weeks; CHD: 32.0 [30.9–32.9] weeks). Of the fetuses with CHD, 16 had coarctation of the aorta, six had transposition of the great arteries, six had tetralogy of Fallot, five had pulmonary atresia, three had hypoplastic left heart syndrome, three had a common arterial trunk, three had an interrupted aortic arch, two had critical aortic stenosis, two had an absent pulmonary valve, one had mitral atresia, one had pulmonary stenosis, one had a double outlet right ventricle, one had total anomalous pulmonary venous drainage and one had an unbalanced atrioventricular septal defect.

### Placental and Fetal Brain T2* Values

**FIGURE 4:**
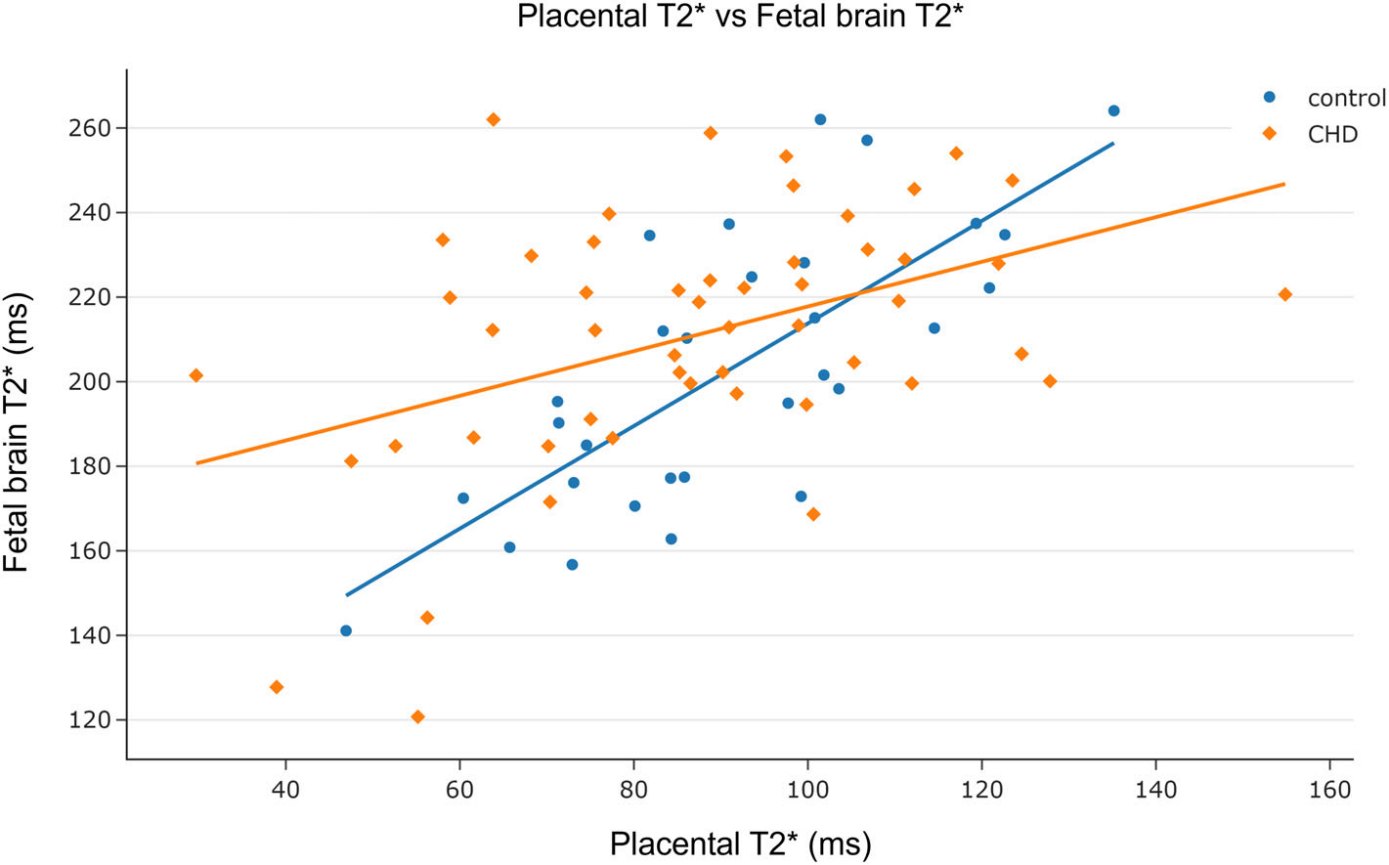
The relationship between placental and fetal brain T2*, after adjusting for GA at scan (partial Spearman's correlation): Entire cohort *⍴* = 0.46, *P*_FDR_ <0.001; control fetuses *⍴* = 0.61, *P*_FDR_ <0.001; CHD *⍴* = 0.36, *P*_FDR_ = 0.011. There was no significant interaction effect of group (interaction coefficient = 0.24, *P* = 0.34).

For the entire cohort, there was a significant positive association between placental and fetal brain T2*, after adjusting for GA at scan (*⍴* = 0.46). This significant positive association held for both control fetuses (*⍴* = 0.61) and for those with CHD (*⍴* = 0.36) when assessed independently. There was no significant interaction effect of group (interaction coefficient = 0.24, *P* = 0.34) (Fig. 4).

### Placental and Fetal Brain Characteristics: Changes Over Gestation

**FIGURE 5:**
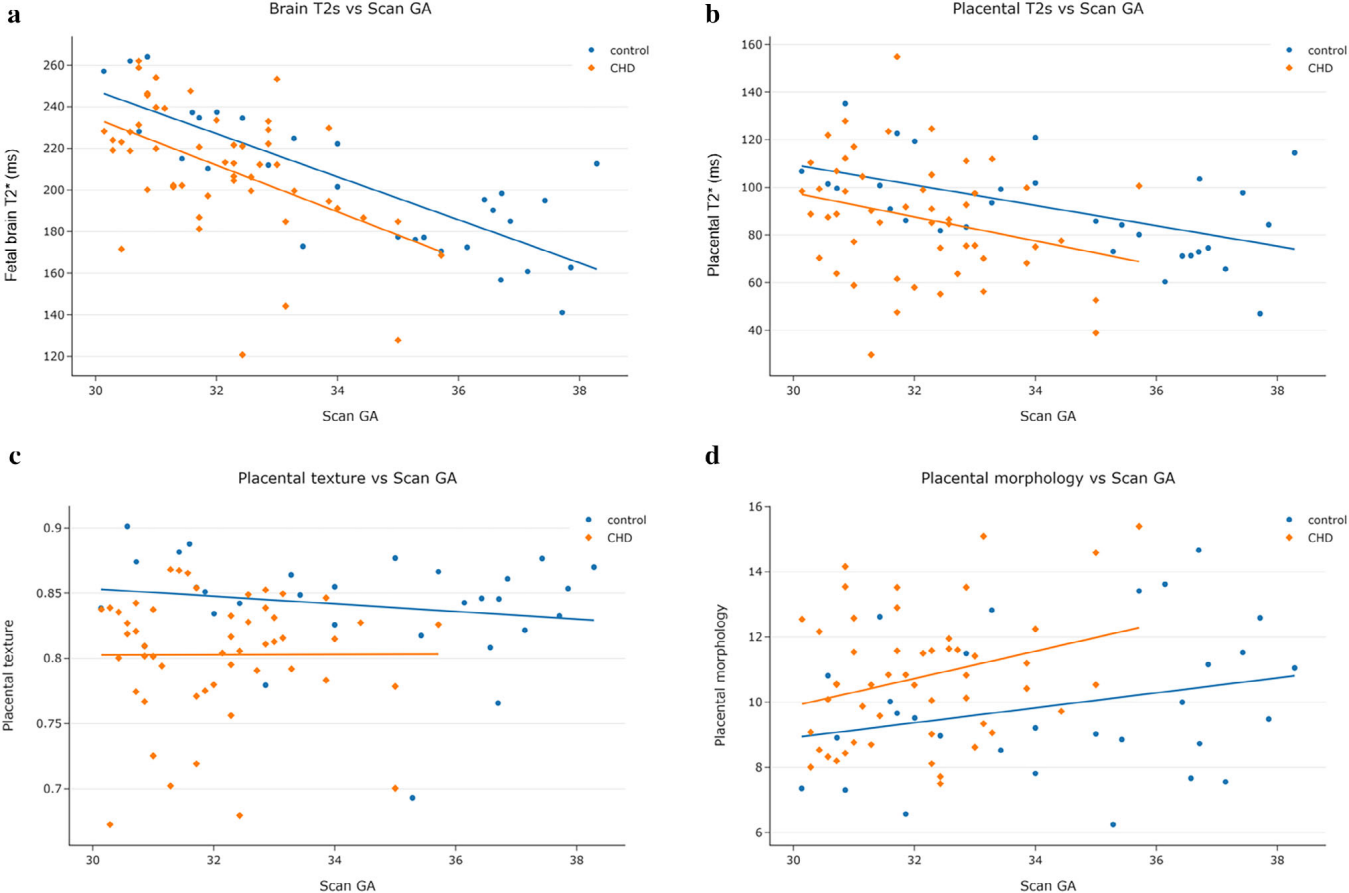
Placental and fetal brain characteristics across gestational age at scan for control fetuses (blue) and those with CHD (orange) that show significant differences between the two groups: (**a**) Fetal brain T2*, (**b**) placental T2*, (**c**) placental texture (with lower values corresponding to more heterogeneous tissues and higher values to more homogeneous tissues), and (**d**) placental morphology (with lower values representing thinner, more uniformly shaped placentas and higher values representing bulkier, less uniformly shaped placentas).

The correlation between placental and fetal brain characteristics with GA for the entire cohort and for both the control and CHD groups are shown in Table 1, Fig. 5, and Fig. S1 in the Supplemental Material. For the entire cohort, placental and fetal brain T2* both showed a significant negative correlation with GA at scan (Placental T2* *⍴* = −0.65, Fetal brain T2* *⍴* = −0.32). Placental volume increased significantly with advancing GA (*⍴* = 0.26). There was no significant association between placental texture (*⍴* = 0.012, *P* = 0.30), maximum thickness (*⍴* = 0.31, *P* = 0.27) or morphology (*⍴* = 0.12, *P* = 0.28) and GA at scan.

**TABLE 1:** Correlation Between Placental and Fetal Brain Measures With Gestational Age

	All (N = 81)	Control (N = 30)	CHD (N = 51)
Fetal brain T2* (msec)	***⍴* = −0.65, *P* < 0.001**	***⍴* = −0.77, *P* < 0.001**	***⍴* = −0.52, *P* < 0.001**
Placental T2* (msec)	***⍴* = −0.32, *P* = 0.004**	***⍴* = −0.51, *P* = 0.004**	***⍴* = −0.28, *P* = 0.03**
Placental volume (mm^3^)	***⍴* = 0.26, *P* = 0.018**	*⍴* = 0.33, *P* = 0.079	*⍴* = 0.21, *P* = 0.14
Placental texture (GLCM correlation)	*⍴* = 0.012, *P* = 0.30	*⍴* = −0.18, *P* = 0.36	*⍴* = −0.056, *P* = 0.67
Maximum placental thickness (mm)	*⍴* = 0.31, *P* = 0.27	*⍴* = 0.21, *P* = 0.27	*⍴* = 0.20, *P* = 0.16
Placental morphology	*⍴* = 0.12, *P* = 0.28	*⍴* = 0.24, *P* = 0.20	*⍴* = 0.20, *P* = 0.16

Results in bold are significant at *P* < 0.05.

### Placental and Fetal Brain Characteristics: CHD vs. Control

Results comparing placental and fetal brain characteristics between CHD and control fetuses are shown in Table 2 and as scatter plots in Fig. 5 and Fig. S1 in the Supplemental Material. After adjusting for GA at scan, placental T2* and fetal brain T2* values were significantly reduced in the CHD group (mean [±SD] placental T2*: control = 97 [±24] msec, CHD = 83 [±23] msec; mean [±SD] fetal brain T2*: control = 218 [±26] msec, CHD = 202 [±25] msec), but there was no significant difference in placental volume (mean [±SD] placental volume: control = 562 (±208) mm^3^, CHD = 608 (±200) mm^3^, *P*_FDR_ = 0.37). Placental texture (GLCM) was significantly lower in the CHD group compared to the control group (median [IQR] placental texture: control = 0.84 [0.83–0.87], CHD = 0.80 [0.78–0.84]), suggesting placentas had a more heterogeneous appearance in the CHD group. Placental morphology was significantly higher in the CHD group compared to the control group (mean [±SD] placental morphology: control = 9.9 [±2.2], CHD = 10.8 [±2.0]), suggesting placentas were bulkier and/or less uniformly shaped in the CHD group. There was no significant difference in maximum placental thickness between groups (mean [±SD] maximal placental thickness: control = 38.7 [±8.2] mm, CHD = 40.5 [±8.1] mm, *P*_FDR_ = 0.37).

**TABLE 2:** Comparisons of Demographics and Placental and Fetal Brain Characteristics Between CHD and Control Fetuses

	Control (N = 30)	CHD (N = 51)	*P*_FDR_ *
Gestational age (GA) at scan (weeks)	34.5 (31.9–36.7)	32.0 (30.9–32.9)	**<0.001**
Maternal age at scan (years)	34.5 (31.1–37.2)	32.2 (29.7–36.2)	0.30
Fetal brain T2* (msec)†	218 (±26)	202 (±25)	**0.027**
Placental T2* (msec)†	97 (±24)	83 (±23)	**0.027**
Placental volume (mm^3^)†	562 (±208)	608 (±200)	0.37
Placental texture (GLCM correlation)	0.84 (0.83–0.87)	0.80 (0.78–0.84)	**0.003**
Maximum placental thickness (mm)	38.7 (±8.2)	40.5 (±8.1)	0.37
Placental morphology	9.9 (±2.2)	10.8 (±2.0)	**0.018**

* *P*‐value for comparing GA at scan and maternal age at scan between groups derived from Mann–Whitney *U*‐test. *P*_FDR_‐values for comparing fetal brain T2*, placental T2* and placental volume between groups all derived from ANCOVA, with GA at scan included as covariate. *P*_FDR_‐values for comparing placental texture, maximal placental thickness and placental morphology between groups derived from ANOVA with no covariates. Results in bold are significant.

^†^ Values presented are estimated marginal means, derived from a general linear model after adjusting for GA at scan.

### Comparing Placental T2* and Placental Texture

**FIGURE 6:**
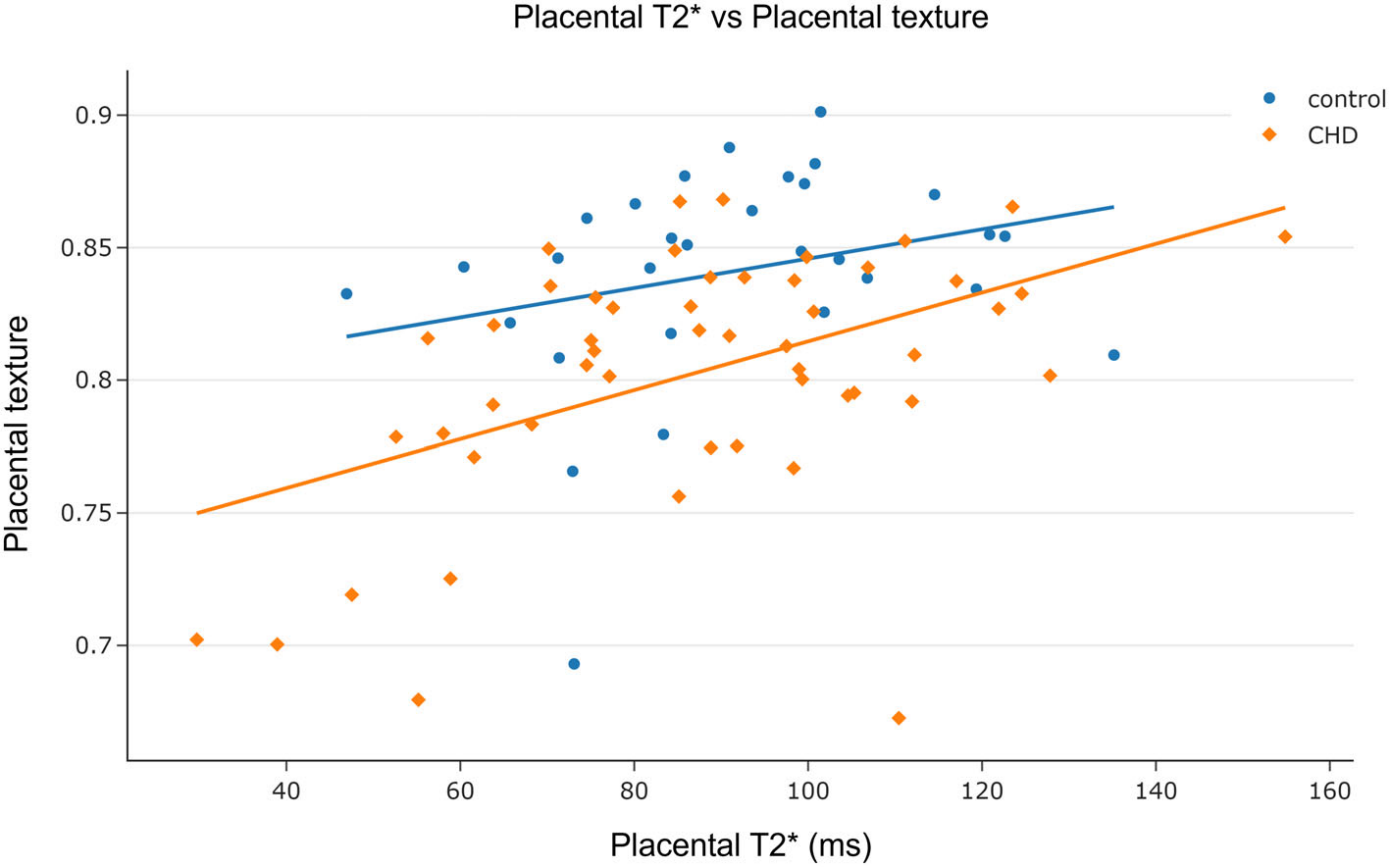
The relationship between placental T2* and placental texture, after adjusting for GA at scan (partial Spearman's correlation): Entire cohort *⍴* = 0.46, *P* < 0.001; CHD fetuses *⍴* = 0.51, *P* < 0.001; control fetuses *⍴* = −0.21, *P* = 0.28. There was no significant interaction effect of group (interaction coefficient = −0.003, *P* = 0.55).

For the entire cohort, there was a significant positive association between placental T2* and placental texture, after adjusting for GA at scan (*⍴* = 0.46). Placental T2* was significantly positively associated with placental texture in the CHD group (*⍴* = 0.51) but not in controls (*⍴* = −0.21, *P* = 0.28) (Fig. 6). There was no significant interaction effect of group (interaction coefficient = −0.003, *P* = 0.55).

## Discussion

In this single‐center, single field‐strength retrospective study, placental and fetal brain T2* values in healthy fetuses and those with CHD were assessed using a single‐shot multi‐echo EPI acquisition and an automated analysis approach. A significant positive correlation between placental and fetal brain T2* was identified, suggesting that placental and fetal brain oxygenation are related, both in healthy fetuses and those with altered cardiovascular physiology. Quantitative measures of placental texture and morphology that are significantly altered in CHD were also identified and showed that placental T2* and placental texture are significantly correlated in fetuses with CHD but not in healthy control fetuses. These findings all support the hypothesis that placental development is altered in CHD.

Both placental and fetal brain T2* values showed a significant decrease over GA, both in healthy fetuses and those with CHD. These findings are in agreement with those from other imaging studies exploring the change in T2* values over gestation in the fetal brain^3^, ^7^ and placenta in normal pregnancies,^6^, ^24^ as well as in the fetal brain in CHD.^15^, ^16^ Similar to these previous studies, the rates of decline in fetal brain T2* over gestation were comparable in healthy controls and fetuses with CHD. The spatial resolution of the EPI scans used here (2.5 mm isotropic) does not allow as accurate measurement of placental and fetal brain volumes as previously published turbo‐spin‐echo based data, which are often implemented specifically for volumetric analyses.^17^, ^18^ Furthermore, while CSF filled spaces of the fetal brain, including the lateral ventricles, cavum septum pellucidum, third and fourth ventricles, and extracerebral CSF were excluded from the quantitative T2* analysis by thresholding, these voxels are included in the automatically generated intracerebral masks. The considerable cortical folding of the fetal brain that is evident in the third trimester also leads to considerable partial voluming effects at these resolutions. For these reasons, volumes derived from the binary intracranial masks in this study were not analyzed.

The exact reasons behind why placental and fetal brain T2* values decrease over gestation remain not fully understood. Direct measurements of the oxygen content of blood in the umbilical vessels have been shown to decrease over gestation.^26^ Some have proposed that for the placenta this relationship results from increased extraction of oxygen from the intervillous space due to increasing fetoplacental metabolic demand, or morphological changes that may alter MRI signal intensity,^27^ which is somewhat supported by the finding that placental texture was only significantly related to MRI signal intensity (T2* values) in fetuses with CHD. For the fetal brain, decreases in T2* values with advancing gestation may be related to changes in tissue composition and structure, including decreasing water density.^7^ As expected, a significant increase in placental volume over gestation was identified, in keeping with other studies using T2* MRI to assess this.^28^ Although no significant association between maximum placental thickness and GA was found, this is to be expected given how much this measurement varies with both placental position within the uterus and fetal position at the time of the MRI.

Significant reductions in placental and brain T2* values in fetuses with CHD compared to controls were also identified, suggesting both cerebral and placental oxygenation are impaired in CHD fetuses. The finding of a reduction in cerebral oxygenation in fetuses with CHD is consistent with that reported in previous work.^15^, ^16^ Importantly, contemporaneously acquired measures of placental oxygenation in the same fetuses were used to show that this reduction in oxygenation throughout the whole brain is significantly correlated with the degree of reduction in placental function.

Reduced placental oxygenation in CHD has previously been reported,^22^ although this is the first time this measure has been compared to cerebral T2* measures. Only fetuses with postnatally confirmed, rather than antenatally suspected CHD, were included, and cases with maternal pathology associated with placental dysfunction, such as hypertensive disorders of pregnancy were excluded. Reductions in placental T2* values have been linked to an increased incidence of vascular malperfusion lesions on placental histological examination^29^ suggesting that this reduction in placental T2* may be related to the increased rate of histological abnormalities seen in CHD.^21^, ^30^

Based on the assumption that the oxygenation of blood flowing into the basal or maternal side of the placenta is unaffected in CHD, it follows that the reduction in placental oxygenation reported here is related to altered haemodynamics on the chorionic side of the placenta. This is supported by evidence from other studies showing that fetal‐placental flows are altered in certain types of CHD^31^ and lends credence to the hypothesis proposed by others that altered villous maturation, consistent with changes in placental microvasculature, could be preventing maximal oxygenation of fetal blood in CHD.^23^ However, we were not able to directly test these hypotheses with this work, which therefore remain speculative. Future studies could attempt to address these issues by measuring directly or inferring the oxygenation of blood flowing into and out of the placenta in CHD.

Shared genetic pathways involved in development of the placenta and fetal heart^32^ and the fetal heart and brain^33^ may also play a role. There also remains the need to consider the underlying cardiovascular physiology when attempting to understand the complex interplay between cerebral and feto‐placental hemodynamics, oxygenation, and placental function.

Several objective measures of placental structure on T2* MRI data that are significantly different between CHD and control fetuses were identified. These may serve as useful imaging biomarkers of altered placental development in CHD. Previous studies have identified reductions in MRI texture‐based measures of fetal organs and the placenta in fetuses with FGR when compared to healthy fetuses.^34^ The finding that GLCM correlation is higher in T2* placental MRI data from healthy pregnancies than those with CHD suggests that on a scale of millimeters the images of the placenta may be more homogenous and has a more consistent pattern of voxel intensities in healthy pregnancies compared to those affected by CHD. These findings reflect those reported in some of the earliest work using T2* MRI to describe how placental structure appears subjectively more homogenous when placental oxygenation is higher, following the administration of maternal oxygen (hyperoxia), than during normoxia.^35^ As well as being related to reduced oxygenation within placental tissue in CHD, this finding might also reflect microstructural or microvascular changes that occur in the placenta in CHD, such as changes in villous structure.^36^

There was a significant difference in morphology, suggesting that the placenta in CHD has a less uniform, disc‐like shape than in normal pregnancy. This is supported by findings from histopathological studies which have reported gross morphological changes in the placenta in CHD.^20^ Despite this, and in accordance with other studies,^37^ no significant difference in placental volume or maximum thickness were identified between control and CHD placentas.

A key strength of this paper is the use of an efficient acquisition obtaining all required data in under 60 seconds. The use of single‐shot multi‐echo EPI freezes motion within each slice, rendering T2* fitting results robust against fetal and maternal motion. A whole‐uterus coronal acquisition was chosen to further facilitate the acquisition and forgo the need for any specialist planning, hence reducing barriers for clinical translation. This approach allows this technique to be added to any fetal MRI scan.

This efficient acquisition is paired with a fully automatic analysis approach allowing calculation of whole organ measures, which is notably different from previous approaches including only selected slices in the brain and placenta.^3^, ^15^, ^35^ This is of particular relevance given the level of observed spatial heterogeneity, calling for a comprehensive characterization of both organs.

The techniques used to assess placental texture and structure were whole‐placental measures, and therefore did not enable assessment of localization of these changes, eg, in the center of lobules close to the mouth of the spiral arteries, or with a pronounced difference between the placental center (defined as close to the cord insertion) and the periphery.^24^ While this is likely to offer improved insight into whole‐placental oxygenation than some previous studies that have used a limited number of manually selected slices to derive T2* values, future work would benefit from being able to measure T2* values within different regions of the placenta, particularly as different compartments are likely to have different functional properties.^38^ Similarly for the brain, assessment was limited to whole brain values, but future work using emerging tools that are capable of parcellating the fetal brain could assess regional alterations in brain T2* values,^39^ particularly since other work has shown that there is likely to be a large variation in T2* values from different areas of the fetal brain.^7^, ^8^

### Limitations

This study was performed in a single center, using a single magnet, vendor, and field strength (1.5 T), and includes a relatively small cohort, which may limit how widely the results can be interpreted. There was a significant difference in the GA at which fetuses in the CHD and control groups were imaged, although this was accounted for in statistical analyses. Future larger age‐matched groups may help to fully characterize the relationships explored here. This study evaluated a limited and heterogenous CHD sample. Further studies with larger numbers are needed to assess the effects of specific CHD diagnoses on placental and fetal brain T2* measures. Furthermore, the GA range studied here was limited to the third trimester as this is the age at which current clinical MRI examinations are undertaken at the institution where this study was performed.

## Conclusion

T2* MRI was used to show there is a direct association between placental and fetal brain oxygenation in both healthy controls and fetuses with CHD. Fetuses with CHD have evidence of decreased placental and cerebral tissue oxygenation. Placental texture on T2* MRI is also significantly altered in CHD, the extent of which relates to the underlying oxygenation of placental tissue.

## Supporting information


**Figure S1:** Placental characteristics across gestational age at scan for control fetuses (blue) and those with CHD (orange) that show no significant differences between the two groups: (A) Placental volume. (B) Maximum placental thickness.
