# Supplementary figures and images for: T2*‐Relaxometry MRI to Assess Third Trimester Placental and Fetal Brain Oxygenation and Placental Characteristics in Healthy Fetuses and Fetuses With Congenital Heart Disease
Source: J Magn Reson Imaging. 2024 Jul 12;61(3):1246–55. doi: 10.1002/jmri.29498 (PMC11803691; doi:10.1002/jmri.29498)

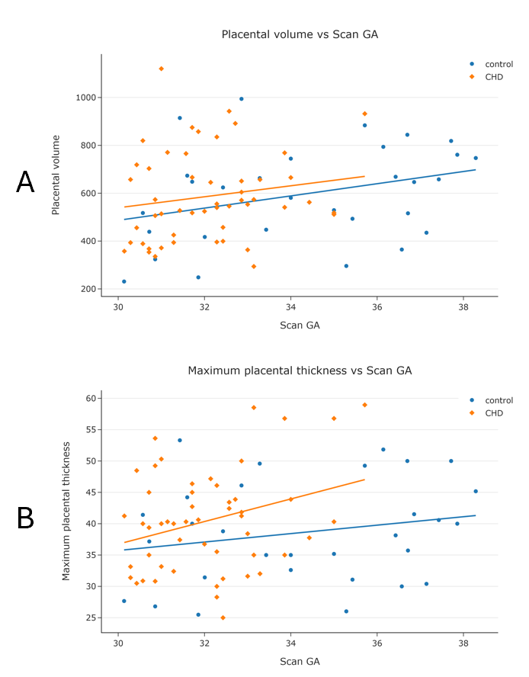

Supplement: Supplementary file 1 — Figure S1: Placental characteristics across gestational age at scan for control fetuses (blue) and those with CHD (orange) that show no significant differences between the two groups: (A) Placental volume. (B) Maximum placental thickness. [file JMRI-61-1246-s001.tif]
